# Influence of Cobalt on the Properties of Load-Sensitive Magnesium Alloys

**DOI:** 10.3390/s130100106

**Published:** 2012-12-21

**Authors:** Christian Klose, Christian Demminger, Gregor Mroz, Wilfried Reimche, Friedrich-Wilhelm Bach, Hans Jürgen Maier, Kai Kerber

**Affiliations:** Institut für Werkstoffkunde (Institute of Materials Science), Leibniz Universität Hannover, An der Universität 2, D-30823 Garbsen, Germany; E-Mails: demminger@iw.uni-hannover.de (C.D.); mroz@iw.uni-hannover.de (G.M.); reimche@iw.uni-hannover.de (W.R.); bach@iw.uni-hannover.de (F.-W.B.); maier@iw.uni-hannover.de (H.J.M.); kerber@iw.uni-hannover.de (K.K.)

**Keywords:** physical sensors, magnetic materials, load-sensitive materials, Villari effect, magnesium, cobalt

## Abstract

In this study, magnesium is alloyed with varying amounts of the ferromagnetic alloying element cobalt in order to obtain lightweight load-sensitive materials with sensory properties which allow an online-monitoring of mechanical forces applied to components made from Mg-Co alloys. An optimized casting process with the use of extruded Mg-Co powder rods is utilized which enables the production of magnetic magnesium alloys with a reproducible Co concentration. The efficiency of the casting process is confirmed by SEM analyses. Microstructures and Co-rich precipitations of various Mg-Co alloys are investigated by means of EDS and XRD analyses. The Mg-Co alloys' mechanical strengths are determined by tensile tests. Magnetic properties of the Mg-Co sensor alloys depending on the cobalt content and the acting mechanical load are measured utilizing the harmonic analysis of eddy-current signals. Within the scope of this work, the influence of the element cobalt on magnesium is investigated in detail and an optimal cobalt concentration is defined based on the performed examinations.

## 1. Introduction

Magnetic magnesium alloys can be utilized as load-sensitive materials with sensory properties because they enable an online-measurement of the instantaneous mechanical loads to which a structural component is subjected during service. With the aid of measured loading history data, the component's resulting fatigue life can be derived and subsequent generations of components can be optimized [1]. In mechanical engineering, the use of strain gauges to measure mechanical loads is state of the art. A major limitation of traditional strain sensors, however, is the locally restricted measurement range, as only local strains directly at the measuring position can be detected. Furthermore, the strain gauges and their electrical contacts are typically applied to the component's surface and thus might be exposed to mechanical damage caused, for example, by external impacts during the use of the component. If the component has material-inherent magnetic properties it can serve as a load sensor itself, because in this case the loading information can be collected with the magnetic material. The application of external mechanical forces to a ferromagnetic material temporarily changes the magnetic susceptibility due to the reversible deformation of the material's crystal lattice [2]. This magnetoelastic effect (also known as Villari effect) is thermodynamically inverse to magnetostriction and leads to a macroscopic change of the magnetic properties [3], which can be monitored by methods of non-destructive component testing, such as the harmonic analysis of eddy-current signals. The application of magnetoelastic force and torque sensors has been reported in literature, e.g., [4,5].

Common ferromagnetic materials are ferritic steels, cobalt and nickel alloys, whereas light metals are paramagnetic. The overall aim in the development of magnetic magnesium alloys is to obtain a lightweight material with inherent load-sensitive abilities. By alloying pure magnesium or magnesium alloys with ferromagnetic elements, particularly cobalt, and ferromagnetic compounds based on samarium-cobalt, several magnesium alloys were produced which exhibited measurable magnetic properties [6–8]. However, in the magnesium industry the element cobalt in general is considered as an impurity due to its negative impact on the corrosion behavior and therefore avoided in the production of magnesium and standard magnesium alloys such as AZ91 [9–12]. This corrosion-accelerating effect is usually related to the existence of undissolved cobalt particles in the microstructure of the magnesium alloy which result from the low solubility of cobalt in magnesium and hence the formation of active cathodic sites [13]. Apart from the frequently described inferior corrosion resistance of cobalt-containing magnesium alloys, little is known about the various other effects caused by cobalt as an alloying element. Some authors reported that small additions of cobalt result in an increase of the strength values of magnesium [14] or magnesium-neodymium alloys [15]. Regarding the magnetic properties of magnesium-cobalt materials, the intermetallic compound MgCo_2_ was found to be ferromagnetic below 321 K [16,17].

One important objective in the development of load-sensitive magnesium alloys is the production of an actual technical alloy in a casting process which is difficult to accomplish due to the high melting point (1,495 °C) and low solubility of cobalt in magnesium. This requirement demands a complete dissolution of the cobalt component in the magnesium melt in order to achieve a homogeneously distributed multiphase microstructure. According to the literature [16,18], a maximum cobalt concentration of 4.5 weight percent (wt.%) is appropriate in order to prevent the formation of undissolved cobalt particles in the magnesium matrix. The microstructure and the suitable magnetic properties of a die-cast binary magnesium-cobalt alloy were briefly introduced by the authors in a previous publication. Here, largely eutectic structures of the cobalt-rich precipitations were observed in images recorded with the scanning electron microscope (SEM) [8]. The specific aim of the present work is to broaden the understanding of the influence that varying amounts of cobalt wield on the characteristics of magnesium-cobalt sensor alloys. In order to qualify these alloys as load-sensor materials, the magnetic properties as well as the microstructures depending on the cobalt concentration and the applied mechanical load are in the focus of this work. After a short presentation of the production process, details on the microstructure, the magnetic and the mechanical properties of magnetic magnesium alloys are discussed in the subsequent sections.

## 2. Experimental Section

For the investigations in this study, five binary Mg-Co alloys with a nominal Co concentration of 1 to 5 wt.% were produced by die casting using technically pure Mg as base material (*cf.*Table 1 for the materials used). Pure Mg specimens were also produced with the same casting parameters for comparison of the magnetic and mechanical properties.

In order to facilitate the dispensation of the ferromagnetic powder in the Mg melt and to avoid floating, 40 wt.% of pure Co powder (particle size < 150 μm) were blended with 60 wt.% of pure Mg powder (particle size < 60 μm) by means of a laboratory powder mixer of the type MP-6 (Biomation, Jugenheim, Germany). The device was equipped with a V-shaped mixing container (volume 1.5 L) and was set to reverse the direction of rotation every 120 s throughout the mixing duration of 150 min. For best results, the mixing container was two-thirds full. Subsequently the powder mixture was compacted to billets with an approximate diameter of 30 mm and a length of 280 mm using a cold-isostatic dry-bag pressing process with a maximum pressure of 2,000 bar which was performed by the company Loomis Products (Kaiserslautern, Germany). After machining to a diameter of 29 mm, the powder billets were extruded to rods (Ø 6 mm) at a temperature of 200 °C using a laboratory extrusion press and a maximum force of 500 kN.

During the subsequent casting experiments the pure Mg melt was inoculated with 1–5 wt.% of Co utilizing the extruded Mg-Co powder rods. The magnetic Mg alloys were manufactured by means of a die casting method using a resistance-heated K4/10 furnace (Nabertherm, Lilienthal, Germany) in a shielding-gas atmosphere (N_2_ + 0.3% SF_6_) and by employing a boron nitride coated, unalloyed steel crucible. The basic material was melted and held at a temperature of 730 °C. At the beginning of the stirring process (45 min at 300 min^−1^), the extruded Mg-Co powder rods were introduced into the melt. The melt was cast into graphite-coated steel molds (Ø 22 mm × length 250 mm) with horizontal feeders which were preheated to 350 °C.

A suitable method for an online-monitoring of the Mg-Co alloys' load-dependent magnetic properties is the harmonic analysis of eddy current signals which is therefore used in this work. A sinusoidal excitation voltage is generated and fed to the excitation coil integrated into the eddy current sensor which generates an alternating magnetic field via the excitation current. The alternating magnetic field is designated as the primary field and produces the magnetic reversal processes and eddy currents which are dependent on the material's influencing variables such as, for example, the formation of the microstructure or the stress state. By means of the eddy currents and the magnetic reversal processes, a secondary field is produced. With the aid of the measuring coil, the signal difference between the primary and the secondary field is measured in comparison to the measuring signal in air. Further details are given in [19]. The harmonics' measured values, computed by a Fast-Fourier-Transformation, provide information about the instantaneous material condition and the lattice distortions as a consequence of the forces acting on the Mg-Co specimens.

The described method was used for a preliminary determination of the magnetic properties of machined cylinder specimens (Ø 18 mm × length 20 mm) from the Mg-Co castings. Furthermore, cyclic loading tests using a universal testing machine of the type Z010 (Zwick Roell, Ulm, Germany) were performed by employing stepwise increasing loads from 200 to 1,400 N in order to test whether correlations can be established between the forces applied to the specimens and their magnetic properties measured by means of the eddy current testing. Threaded cylindrical tensile specimens with a nominal diameter of 6 mm (according to DIN 50125) were machined by turning as test pieces for both the cyclic loading experiments using the Villari effect and the determination of the mechanical strength values in standard tensile tests. Three specimens of each alloy were used in the tensile tests.

For the examination of the Mg-Co alloys' microstructure, SEM investigations were carried out employing the compositional contrast mode (RBSD) of a LEO 1455VP SEM (Carl Zeiss Microscopy, Jena, Germany). This method was used by the authors before in order to distinguish the microstructure constituents of binary Mg-Co alloys [8]. Sections of the Mg-Co castings were metallographically prepared by grinding using SiC abrasive papers up to grade P2500. Afterwards, the specimens were polished to 1 μm using diamond paste and rinsed with ethanol.

Corresponding to the SEM analyses of the Mg-Co alloys, measurements with energy-dispersive x-ray spectroscopy (EDS) employing a Quantax EDS system (Bruker, Billerica, MA, USA) and a measuring time of 120 s each were performed on the specimens in order to determine the alloys' overall Co concentrations. The SEM investigations were supplemented with phase analyses by means of XRD measurements using an X'Pert MPD system (PANalytical, Almelo, the Netherlands). The measurement was carried out using Cu_Kα_ radiation in the range of 5–110° 2Θ with an increment of 0.02° and a measuring time of 16 s per step. An evaluation of the measured diffraction patterns was performed by means of the XRD database PDF-2 ver. 2010.

In addition, micro tensile specimens with a rectangular cross section of 3 × 2 mm in the taper and a length of 60 mm were manufactured by continuous-wire electro discharge machining and polished as described above. These samples were examined in-situ with the aid of a SEM-fitted tensile/compression module (Kammrath & Weiss, Dortmund, Germany). At several stages during the tensile test, SEM images of the alloy's microstructure were recorded.

## 3. Results and Discussion

In the subsequent sections, the Mg-Co alloys' nomenclature includes the corresponding nominal concentrations of Mg and Co in weight percent. For example, Mg96Co4 means pure Mg alloyed with 4 wt.% of pure Co. The results presented in the following chapters are mainly focused on the alloy Mg96Co4 due to its advantageous properties.

### 3.1. Magnetic Properties

**Figure 1.**
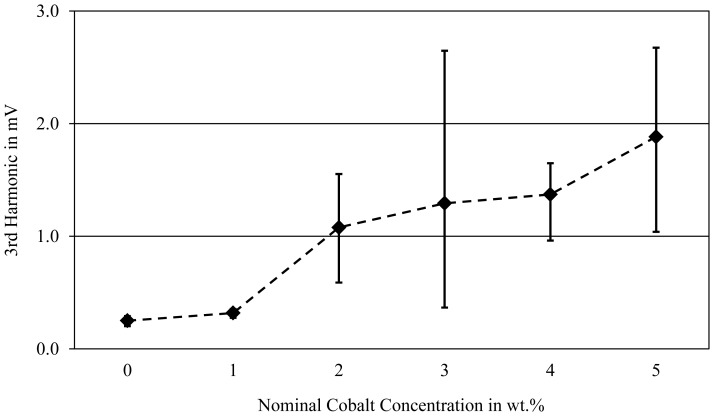
Amplitudes of the 3rd harmonic of the binary Mg-Co alloys' cylinder specimens; eddy current ring sensor Ø 20 mm, test frequency 1.6 kHz.

The magnetic properties of the five binary Mg-Co alloys were examined with the aid of a harmonic analysis of eddy current signals. For a material's magnetic properties the 3rd harmonic is of major significance. It will be close to zero if the tested material is not ferromagnetic; higher values indicate better magnetic properties. In Figure 1 a screening of the produced Mg-Co alloys' average 3rd harmonics in their initial condition is presented by means of measurements on the cylinder specimens. As one might expect, a higher Co concentration leads to greater amplitudes of the 3rd harmonic and thus to stronger magnetic properties. While the alloy with 1 wt.% Co exhibits very low amplitudes which are on the same level as pure Mg, a significant increase is achieved with alloys containing at least 2 wt.% Co. The alloy Mg96Co4 shows an optimal trade-off between the average amplitude and the scatter of the values.

**Figure 2.**
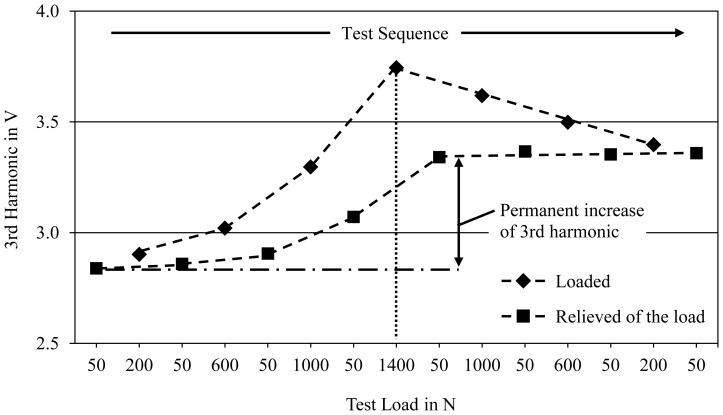
Amplitudes of the 3rd harmonic of Mg96Co4 measured in loading tests; eddy current ring sensor Ø 10 mm, test frequency 1.6 kHz.

In order to qualify the alloys' material-inherent load sensor abilities, tensile specimens manufactured from all of the Mg-Co castings were subjected to cyclic loading measurements. As the results show, the magnetic properties of alloys containing at least 2 wt.% Co are sufficient to distinguish the values of the 3rd harmonic between the loaded and the relieved condition. Moreover, an obvious dependency exists between the applied mechanical loads and the measured amplitude values which is demonstrated in Figure 2 by means of the alloy Mg96Co4. Following the test sequence from left to right, every dot is the average result of three consecutive measurements at the same test force. While a force of 200 N leads to a slightly greater amplitude than in the initial state, the highest load in this experiment (1,400 N) causes the 3rd harmonic to increase by 30%. These magnetic Mg alloys can therefore be classified as load-sensitive materials.

After each step the specimen was relieved of the load (50 N) with the amplitudes of the 3rd harmonic dropping back to a lower value. There is however a gradual increase of the amplitudes in the unloaded condition. After reaching the maximum test load, the 3rd harmonic's measured values remain constantly on the higher level. This effect is presumably connected with the low yield strengths of the binary Mg-Co alloys. Concluding from results of the tensile tests which are discussed in Section 3.3 and the sample's cross-section (∼30 mm^2^), the alloy's yield strength is exceeded at a load of approx. 1,000 N. The beginning plastic deformation by uniaxial strain affects the microstructure of the magnetic material and leads to a permanent change of the magnetic properties.

**Figure 3.**
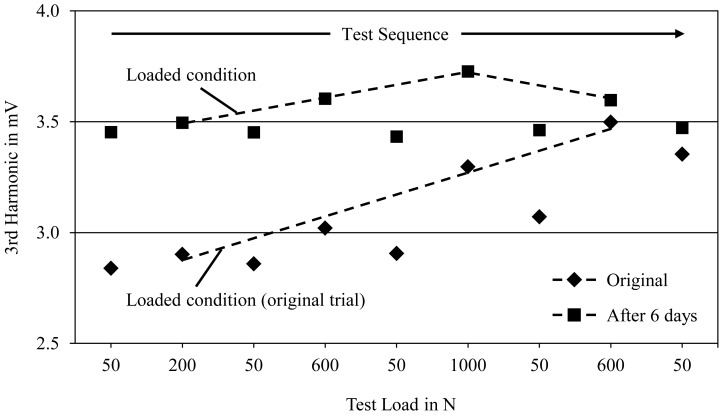
Amplitudes of the 3rd harmonic of Mg96Co4 measured in loading tests which were performed twice on the same tensile specimen with a time interval of 6 days; eddy current ring sensor Ø 10 mm, test frequency 1.6 kHz.

In a first step in order to examine the reproducibility of this effect, the load test was repeated after a time interval of 6 days utilizing the identical tensile specimen as before. Note that the test rig was disassembled in the meantime and set-up again for the repeated trial. An overview of the results compared with the original measurement is given in Figure 3. During the second loading experiment, the 3rd harmonic's measured values are constant around 3.45 mV in the relieved state and hence on a similar level like at the end of the original load test (∼3.4 mV). Furthermore, the load-sensitive behavior of the alloy Mg96Co4 is still present with an increase of 0.28 mV per 1,000 N. Although the gradual and permanent increase of the 3rd harmonic, which could be observed as a result of high loads, still has to be investigated in greater detail, it suggests its use for the detection of local plastic deformation of structural components made from magnetic Mg alloys. In this case, the chance of quantifying a component's overloading by eddy current measurements after the end of its lifecycle would be one great advantage in addition to the sensory behavior of Mg-Co alloys.

### 3.2. Microstructure Investigation and Phase Analysis

**Figure 4.**
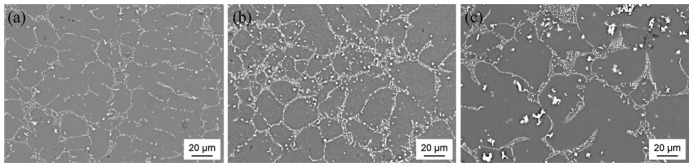
SEM images of the microstructures of binary Mg-Co alloys (RBSD mode): (**a**) Mg97Co3; (**b**) Mg96Co4; (**c**) Mg95Co5.

The following SEM images show sections of three of the binary Mg-Co alloys' microstructures (Figure 4). Due to their higher atomic mass numbers, the Co-rich phases appear as bright structures which are easily distinguished from the Mg matrix. Independent of the global Co concentration, the Co-containing phases exhibit a eutectic structure in all the examined alloys and form a refined, widespread network within the Mg matrix. With the Co content increasing from 3 wt.% (Figure 4(a)) to 4 wt.% (Figure 4(b)), the number of lamellar-arranged precipitations grows and they seem to take up a greater proportion of the material's volume. Furthermore, the hypereutectic alloy Mg95Co5 (Figure 4(c)) shows an increased quantity of agglomerated particles which are not connected to the eutectic phase structure. Most of the agglomerates are surrounded by areas of darker gray-shades which indicate regions of lower density and thus beginning corrosion. These undissolved particles originate from the ferromagnetic Co powder and, in this work, can be observed mainly in alloys with an overall Co concentration of more than 4 wt.%. These results agree with the data given in literature regarding the limited solubility of Co in the Mg melt [16,18] (see also Section 1: Introduction).

In contrast to the authors' previous publications [7], the results of the EDS measurements (Table 2) which were performed in order to determine the overall amounts of Co in the produced Mg alloys show good compliance to the nominal amounts of the ferromagnetic alloying element the Mg melts were inoculated with during the casting process. This can be credited to the use of the extruded powder rods, as they facilitate the alloying process and obviously increase alloying efficiency. For the hypereutectic alloy Mg95Co5, a low amount of silicon was measured which, in this case, can be attributed to the use of SiC-containing abrasive paper during the sample preparation procedure. Also, the measured carbon and oxygen concentrations are within the usual limits of magnesium alloy samples which were prepared by mechanical grinding and polishing.

**Figure 5.**
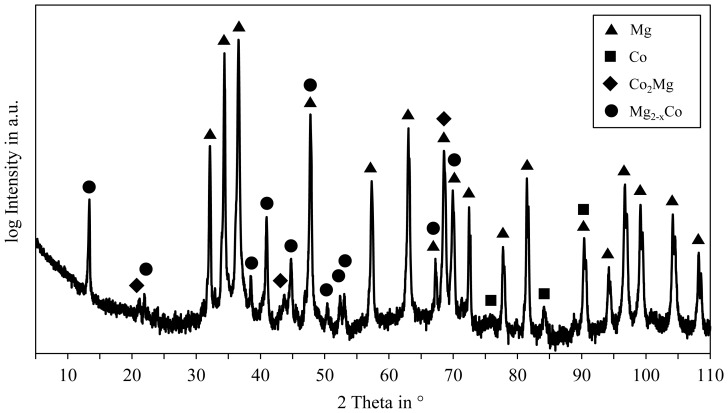
Diffraction pattern of the alloy Mg96Co4 in as-cast state.

The multiphase structure, as can be seen in the SEM images (Figure 4), suggests the existence of primary Mg and Co crystals as well as intermetallic compounds, though few intermetallic phases are possible in the Mg-Co system [20]. The diffraction pattern resulting from the XRD measurement of the Mg96Co4 specimen is shown in Figure 5. As expected, the peaks with the highest intensities belong to the primary Mg crystals occupying the largest part of the sample's surface area. Due to the hexagonal crystal structure of both Mg and Co at room temperature, the reflexes of both metals overlap partially, e.g., at 90° 2Θ. However several peaks were found that cannot be fitted to either Mg or Co but, according to the XRD database used in this work, clearly indicate the existence of intermetallic compounds of the types Co_2_Mg and Mg_2−x_Co in the cast specimen. The exact stoichiometry of intermetallic phases in the Mg-Co system is discussed controversially in literature [16,20], yet their presence together with the eutectic structure proves the dissolution of the Co powder in the Mg melt. The compound Co_2_Mg at least contributes to the macroscopic magnetic properties of these alloys because it is ferromagnetic at room temperature [17].

### 3.3. Mechanical Properties

**Figure 6.**
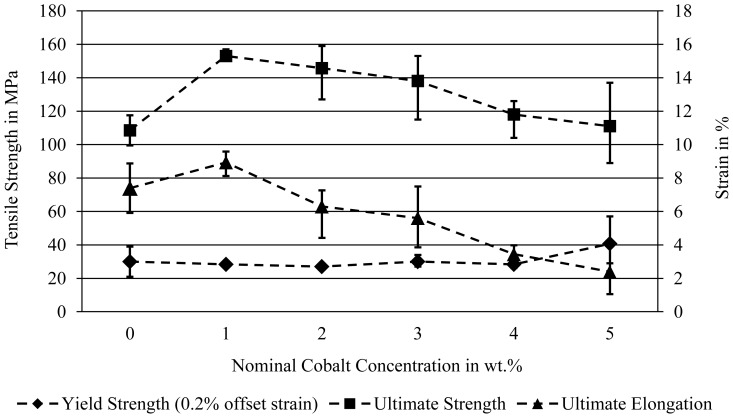
Mechanical properties of binary Mg-Co alloys in the as-cast state subject to the Co content; tensile tests according to ISO 6892-1, threaded tensile specimens DIN 50125 B 6 × 30 (mean values of 3 specimens for each alloy).

Standard tensile tests were performed in order to assess the mechanical strength properties of the produced alloys in the as-cast condition. The results reveal that the influence of the Co addition is reflected mainly in the changes of the ultimate tensile strength (Figure 6). For the alloy containing 1 wt.% Co, an increase of 40% (153 MPa) compared with pure Mg (108 MPa) is observed. With increasing amount of Co, the tensile strength is reduced gradually; the alloy Mg95Co5 has the same tensile strength as pure Mg only. The ultimate elongation tends to correspond with the tensile strength and ascends to approx. 20% higher values in case of the alloy Mg99Co1 (8.9% elongation) but drops to a very low level at the higher Co concentrations (2.4%). In contrast, the yield strength remains almost constant around 30 MPa with the exception of the hypereutectic alloy Mg95Co5 which reaches 40 MPa. Apart from the slightly higher global Co concentration the large number of undissolved Co particle agglomerates is the main difference in the microstructure of Mg95Co5 compared with the other alloys. They result from an oversaturation of the Mg melt with Co and might to some extent have the same effect as a particle reinforcement.

**Figure 7.**
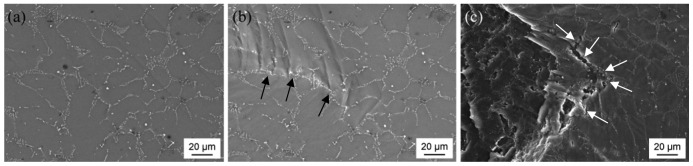
SEM investigation of the microstructure of the alloy Mg96Co4 during the in-situ tensile test: (**a**) specimen 1 in unloaded state; (**b**) specimen 1 loaded with 630 N; (**c**) fracture in specimen 2 after failure.

For an explanation of the measured tensile strengths the behavior of the alloy Mg96Co4 during plastic deformation and failure was investigated by means of in-situ tensile tests. In Figure 7 the same area is displayed in its initial state (Figure 7(a)) and under the sample's highest tolerated force (630 N; Figure 7(b)). The arrows in Figure 7(b) indicate a continuous precipitation which seems to act as a barrier preventing the deformation of the adjacent grain. It is evident that mainly the Mg matrix is influenced while the Co-containing phases partly shield the loads. This may lead to increased stresses in a limited number of grains and finally to early failure of the sample.

On a second specimen crack initiation could be observed next to the eutectic phases whereas the cracks propagated along the precipitations (Figure 7(c)). Obviously the Co-rich eutectic phase structure wields a major influence on the strength properties of the binary Mg-Co alloys due to its lamellar morphology and higher hardness than the Mg matrix. Compared with pure Mg (Figure 6), an increasing overall Co content leads to a more brittle behavior which can be attributed to the growing precipitation of Co-rich phases in the microstructure.

## 4. Conclusions

In the present work the production of magnetic Mg alloys based on pure Mg and pure Co was demonstrated by die casting. The aim of this study was to show the influence of Co concentrations between 1 and 5 wt.% on the microstructure and the phase formation as well as on the mechanical and magnetic properties. The results contribute towards a better understanding of the challenges in the production and the various properties of Mg sensor alloys.

Because the solubility of Co in Mg is negligible [16] and hence the ferromagnetic element Co precipitates in a eutectic structure, it is evident that the Mg-Co alloys' magnetic properties must be related primarily to the Co-rich eutectic precipitations that could be observed in the SEM images. XRD analyses confirmed the existence of primary Co phases and suggested the formation of intermetallic compounds between Mg and Co in the microstructure. The magnetic properties strongly depend on the overall amount of Co in the alloy. In particular, the higher-alloyed materials Mg96Co4 and Mg95Co5 show better magnetic properties in the sense of greater amplitudes of the 3rd harmonic. As a consequence, this means a higher sensitivity for the online-measurement of the applied mechanical loads because of the larger range of the measured values between the initial, unloaded state and the highest load. However, the large quantity of undissolved Co agglomerates in the hypereutectic alloy Mg95Co5 indicates an oversaturation with Co which can lead to accelerated corrosion due to the particles' effect as active cathodic sites in the Mg matrix. Hence the authors suggest an optimal Co concentration of 4 wt.% for the production of magnetic Mg alloys.

Regarding the mechanical properties in the as-cast state, the low yield strength of the binary Mg-Co alloys is an obvious problem. The tensile tests revealed that the impact of an increasing Co content shows mainly in the decrease of the tensile strength and the elongation at fracture. On the other hand, a connection between the magnetic properties and the yield strength could be observed during the loading experiments which appears as a gradual and permanent increase of the 3rd harmonic of the eddy current signal. This effect occurred usually as a result of high test loads and thus might be of use for the detection of overloaded parts of structural components without the need for an active permanent online-monitoring of the forces acting on the component.

## 5. Outlook

**Figure 8.**
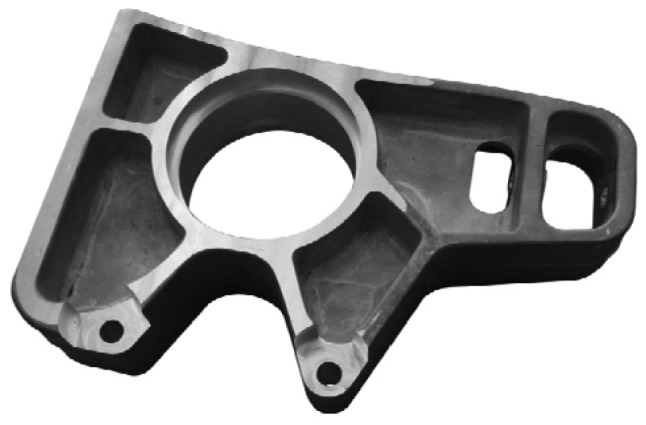
Load-sensitive magnesium wheel carrier from the RP09 race car (Team Horsepower, Leibniz Universität Hannover).

Apart from the high melting points and the poor dissolving behavior of the ferromagnetic alloying elements in Mg the greatest challenge in the development of sensory Mg alloys is to find the optimum between usable magnetic properties and reasonable mechanical properties. The authors already demonstrated the use of Zn as an additional alloying element in low concentrations [7,8] which improves the strength and, to some degree, the castability. In order to examine the interactions of Co and standard alloying elements like Al, Zn and Mn further investigations are currently made which focus on enhancing the mechanical properties in the as-cast state and primarily the yield strength. These results will be published in a future work. Within the scope of this project, the magnetic Mg alloys' load-sensitive behavior will also be demonstrated on actual structural components with a complex geometry. For these tests, the rear wheel carrier of a Formula Student racing car of Leibniz Universität Hannover was chosen and produced by casting (Figure 8).

## Figures and Tables

**Table 1.** Materials used in this study.

**Material**	**Supplier**	**Content in wt.% according to Supplier**							

		**Zn**	**Al**	**Si**	**Mn**	**Co**	**Fe**	**Ca**	**Mg**

Mg ingots	Magnesium Elektron	0.004	0.012	0.012	0.025	-	0.002	0.02	99.92
Mg powder	Ecka Granules	-	-	-	-	-	-	-	99.8
Co powder	Sigma Aldrich	-	-	-	-	99.9	-	-	-

**Table 2.** EDS measurements of overall Co concentrations in binary Mg-Co alloys.

**Alloy**	**Nominal Co Content in wt.%**	**Element concentration in wt.%**				

		**Mg**	**Co**	**C**	**O**	**Si**

Mg97Co3	3	94.71	2.99	1.93	0.37	-
Mg96Co4	4	94.99	3.44	1.57	0.00	-
Mg95Co5	5	92.66	4.47	1.79	0.95	0.12
